# Word- and Text-Level Processes Contributing to Fluent Reading of Word Lists and Sentences

**DOI:** 10.3389/fpsyg.2021.789313

**Published:** 2022-01-10

**Authors:** Sietske van Viersen, Athanassios Protopapas, Peter F. de Jong

**Affiliations:** ^1^Department of Special Needs Education, University of Oslo, Oslo, Norway; ^2^Research Institute of Child Development and Education, University of Amsterdam, Amsterdam, Netherlands

**Keywords:** word reading, sentence reading, fluency, word recognition, serial naming, RAN, vocabulary, syntactic skills

## Abstract

In this study, we investigated how word- and text-level processes contribute to different types of reading fluency measures. We aimed to increase our understanding of the underlying processes necessary for fluent reading. The sample included 73 Dutch Grade 3 children, who were assessed on serial word reading rate (familiar words), word-list reading fluency (increasingly difficult words), and sentence reading fluency. Word-level processes were individual word recognition speed (discrete word reading) and sequential processing efficiency (serial digit naming). Text-level processes were receptive vocabulary and syntactic skills. The results showed that word- and text-level processes combined accounted for a comparable amount of variance in all fluency outcomes. Both word-level processes were moderate predictors of all fluency outcomes. However, vocabulary only moderately predicted sentence reading fluency, and syntactic skills merely contributed to sentence reading fluency indirectly through vocabulary. The findings indicate that sequential processing efficiency has a crucial role in reading fluency across various measures besides individual word recognition speed. Additionally, text-level processes come into play when complexity and context availability of fluency measures increases, but the exact timing requires further study. Findings are discussed in terms of future directions and their possible value for diagnostic assessment and intervention of reading difficulties.

## Introduction

Reading fluently and comprehending text are essential skills in our literate society. Yet, what exactly entails fluent reading is still debated. Definitions of reading fluency show great variation; they range from rather narrow, only considering rate of word recognition (e.g., Ehri and Wilce, 1983), to very wide, encompassing all aspects of reading including comprehension (Samuels, 2006, 2007; see Breznitz, 2006, for an overview). This variation results from a strong divide between studies on underlying processes involved in fluent reading and processes related to comprehension of texts. Both types of studies come from largely separate domains with their own research traditions. Consequently, we have some knowledge about basic word-level processes underlying reading fluency of words (e.g., de Jong, 2011; Protopapas et al., 2013; van den Boer and de Jong, 2015; Zoccolotti et al., 2015; Altani et al., 2018) and about text-level processes involved in the reading fluency of texts (e.g., Fuchs et al., 2001; Jenkins et al., 2003). However, it is still unclear how demands on individual, basic word-level processes underlying reading fluency differ across relevant fluency measures. Moreover, we have limited knowledge about how word-level processes might interact with text-level processes when the complexity of fluency measures changes or context becomes available.

Here, we investigated to what extent basic word- and text-level processes contribute to a variety of reading fluency measures, aiming to better understand the mechanisms underlying fluent reading. We generally adhere to the widely accepted definition of the National Reading Panel [NRP], 2000 stating that reading fluency is “the ability to read a text quickly, accurately, and with proper expression (p. 3–5, see also Hudson et al., 2005; Kuhn et al., 2010). In our study, however, we wish to bring together word-list and text-based metrics of fluency. We therefore omit prosody, which is not relevant for word lists. Our working definition of reading fluency is thus the accurate and rapid reading of a series of words. Specifically, we assessed (a) word-list reading of simple familiar words (i.e., serial word reading rate; covering unrelated short high-frequency words of low difficulty), (b) word-list reading of increasingly difficult unfamiliar words (i.e., word-list reading fluency; as in common tests of “word reading efficiency,” e.g., Torgesen et al., 2012; covering unrelated longer and lower frequency words), and (c) reading fluency of sentences. These fluency measures represent a gradual increase in complexity regarding word length and familiarity as well as context availability. As such, these measures may tap underlying word- and text-level processes differentially, so that differences can be detected in the relations of the three measures to underlying word- and text-level processes.

Word recognition, or processing efficiency of individual words, is one of the main word-level processes related to reading fluency. After all, how rapidly and effortlessly a child can identify single words will largely determine the child’s potential reading speed of series of words. In the development of reading skill, word identification generally starts out as a slow and laborious process in which words are deciphered letter-by-letter using grapheme-phoneme correspondence rules. Repeated successful identification through phonological recoding helps the child to form an orthographic representation of the word (Share, 2008). This representation makes it easier to recognize the word, in larger chunks or as a whole, in future encounters. Eventually, a word is assumed to become part of the child’s sight word vocabulary. The child is then able to recognize the word at a glance, that is, retrieve its pronunciation from memory on seeing the written form (Ehri, 2005, 2014). This gradual change from letter-by-letter decoding to sight word reading has long been considered the key explanation for the development of word-level reading fluency as measured by word list formats. Hence, prediction of reading fluency of word lists by the recognition rate of individual words (as measured by a discrete reading task displaying only one word at a time) should be close to perfect, if individual word recognition were indeed the sole factor underlying the development of the fluent reading of lists of words. Likewise, outcomes on the two kinds of measures should be almost identical. However, multiple studies have shown that this is not the case (e.g., de Jong, 2011; Protopapas et al., 2013; Altani et al., 2020).

Recent research by Protopapas et al. (2013, 2018) suggests that the presence of multiple simultaneously available words in a sequence, as opposed to the display of one single word at a time, is a critical feature that distinguishes word-list fluency tasks from individual word recognition tasks. They hypothesize that sequential processing efficiency (i.e., the ability to process multiple items in a sequence) is an additional ability that is crucial for achieving reading fluency. Sequential processing efficiency is believed to depend on the ‘cascaded’ processing of words or other stimuli. Multiple items in a sequence are processed *simultaneously*, but at different stages: While the first word is articulated, the second word is processed, the third is viewed, and the fourth is previewed, all of which happens largely in parallel (e.g., Protopapas et al., 2013, 2018). Accordingly, sequential processing efficiency specifically taps into the coordination of these processes between multiple items in a sequence. This coordination can only be optimized once individual words are recognized instantly (by sight).

Studies into the nature and measurement of sequential processing efficiency have shown that this skill can be captured by serial rapid automatized naming (RAN) tasks (de Jong, 2011; Protopapas et al., 2013). Naming of digits seems to capture the sequential processing of adjacent items best in relation to reading fluency of word lists, even though other kinds of serial naming tasks target this process as well (e.g., including objects, number words or dice; Protopapas et al., 2018). This may be because the individual elements in serial digit naming tasks are automated to such an extent that they allow unmediated one-chunk processing, closely mimicking reading words by sight (de Jong, 2011; Protopapas et al., 2018, see also Altani et al., 2020). Consequently, the sequential processing of multiple familiar items is what dominates performance in the serial digit naming task.

Reading fluency will require more than individual word recognition and sequential processing efficiency when the words are connected, such as in sentences or texts. Words in sentences and texts are not combined randomly but are connected to each other by supra-lexical elements, structures, and operations. As such, fluent reading of connected text requires semantic and syntactic processing (e.g., Ouellette, 2006; van Silfhout et al., 2015). Previous research has shown that skilled and less skilled readers rely on their knowledge of words and syntactic relations to support word recognition during reading (e.g., West et al., 1983; Nation and Snowling, 1998; Mokhtari and Thompson, 2006). More specifically, identification of anaphoric referents, use of connectives, and semantic probability have been identified as factors that facilitate reading fluency of sentences and texts (Perfetti, 1995; Frisson et al., 2005; Crosson and Lesaux, 2013; van den Bosch et al., 2018). This indicates that basic word-level processes as well as comprehension processes play a role in fluent reading at higher levels of complexity (e.g., Jenkins et al., 2003; Kim et al., 2014). Therefore, in this study we assess the role of receptive vocabulary and syntactic skills as relevant text-level processes across reading fluency measures and investigate their contribution in addition to the individual word-level processes mentioned above.

Regarding the word-list reading of familiar words (i.e., short words of high frequency that are likely to be read by sight), multiple studies have shown that individual word recognition speed is only a moderate predictor of serial word reading rate (Protopapas et al., 2013; Altani et al., 2017, 2020). Additionally, several studies have shown that the relation between individual word recognition speed and serial word reading rate decreases over time (Protopapas et al., 2013, 2018; Altani et al., 2020). The predictive power of individual word recognition skill is weakened as children become more skilled readers and are able to read word lists more fluently (de Jong, 2011; Altani et al., 2020). This indicates that the reading processes underlying reading fluency of word lists change over time and skills other than individual word recognition speed become more important for fluent reading (see also van den Boer and de Jong, 2015).

Indeed, multiple studies have shown that serial digit naming is also a unique predictor of serial word reading rate (van den Boer et al., 2016; Altani et al., 2017, 2018) and explains additional variance beyond individual word recognition speed (de Jong, 2011; Protopapas et al., 2013; van den Boer and de Jong, 2015; Altani et al., 2020). Moreover, the correlation between serial digit naming and serial word reading rate is stable or even increasing over time (de Jong, 2011; Protopapas et al., 2013, 2018; Altani et al., 2018, 2020). This pattern of findings has been observed across orthographies varying in transparency (i.e., Greek, Italian, Dutch, English; Zoccolotti et al., 2013; van den Boer et al., 2016; Altani et al., 2017, 2020; see also Moll et al., 2014; Landerl et al., 2019, on the role of RAN in reading fluency of word lists across orthographies). The same pattern has also been found across different writing systems (i.e., Chinese, Korean; Altani et al., 2017; see also Araújo et al., 2015, for an overview of relevant aspects of the RAN-reading relationship).

For example, in a study on Grade 3 children, Altani et al. (2020) showed that both individual word recognition speed and sequential processing efficiency are important and unique predictors of serial word reading rate. Word recognition contributed slightly more in Greek (in which the word list was composed of simple two-syllable words), whereas sequential processing contributed more in English (using one-syllable words). Combined, both word-level processes explained about 50% of the variance in serial word reading rate. Altani et al. (2017; using the same data for Greek and English) showed that sequential processing efficiency was also the larger contributing factor in Korean and Chinese. This resulted in similar amounts of total explained variance in Korean (51.2%), but much less in Chinese (31.2%). Based on these findings, we consider individual word recognition speed and sequential processing efficiency to be the two main word-level reading processes underlying reading fluency. We know nothing about the role of text-level comprehension processes in relation to serial word reading rate. However, it is unlikely that they greatly influence reading fluency in simple tasks in which words are expected to be read by sight.

Word-lists of increasingly difficult words (i.e., longer words of low frequency that cannot all be read by sight) are frequently used in educational and diagnostic settings. Nonetheless, we know very little about how individual word- and text-level processes affect these fluency measures (e.g., TOWRE; Torgesen et al., 2012). Evidently, individual word recognition speed and sequential processing efficiency are expected to play an essential role. Yet, the fact that not all words can be read by sight in measures of increasing difficulty might make a crucial difference. de Jong (2011) was the first to suggest that sequential processing may capture serial processes both between words and *within* words that are not yet read by sight. Supporting evidence comes from correlations between discrete and serial RAN and word-reading tasks in beginning and more advanced readers. Specifically, in more advanced readers, the strong correlations that are found between serial RAN and *serial* word reading suggest that words are activated in an automated fashion, like single digits; that is, they are read by sight. In beginning readers, however, correlations are strongest between serial RAN and *discrete* word reading tasks. This pattern of correlations suggests that less-skilled readers identify words by processing a series of individual elements, that is, letter-by-letter or letter cluster-by-letter cluster, because they cannot yet read the words by sight (de Jong, 2011; van den Boer et al., 2016; Altani et al., 2018). Sequential processing might thus play a bigger role in word-list reading of increasingly difficult words than in word lists of familiar words. In the latter, sequential processing is restricted to between-word processing, because each single word is assumed to be read by sight. In the reading of a list of increasingly difficult words, however, sequential processing is related to both between- and within-word processing. Surprisingly, the only available data show that correlations of discrete word reading and serial digit naming with word-list reading of familiar and increasingly difficult words are comparable (de Jong, 2011). In addition, the influence of discrete word reading was found to decrease for both types of word lists as children become better readers (de Jong, 2011). That is, there is no evidence that word lists of increasingly difficult words pose increasing demands on sequential processing efficiency, compared to lists of familiar words. This suggests that other factors are more important for individual differences in word-list reading fluency, so that the relative contribution of sequential processing to the total variation is limited.

One potential factor explaining additional variance in word-list reading fluency may be vocabulary knowledge, even though word-list reading fluency cannot really be considered a complex fluency measure in terms of semantic relations between words. A larger vocabulary is reflected in a larger phonological lexicon. This larger phonological lexicon would facilitate the build-up of an orthographic lexicon, because the phonological representation of a word does not have to be acquired (as would be the case in the reading of non-words). This might thus increase the probability that words are read by sight. Alternatively, vocabulary might also play a role in the fast and accurate recognition of the more difficult words of lower frequency that cannot be (fully) read by sight. Multiple studies have shown that vocabulary is generally more strongly related to word reading of irregular words than regular words (Nation and Snowling, 2004; Ricketts et al., 2007; Krepel et al., 2021; but see Ricketts et al., 2016). Also, children with larger vocabularies tend to be better at word reading (see Taylor et al., 2015, for an overview). Ouellette (2006) has looked specifically into the role of vocabulary in word-list reading fluency of increasingly difficult words in French. The findings showed that vocabulary size was an independent predictor of word-list reading fluency and explained unique variance even after accounting for pseudoword decoding. In contrast, research in Dutch has shown that the relation between vocabulary and word-list reading fluency is rather weak (de Jong and van der Leij, 2002; de Jong, 2011). A recent study by Kim (2015) in Korean has looked at the combined influence of individual word- and text-level processes on word-list reading fluency. The results suggested that vocabulary may explain unique variance in word-list reading fluency after sequential processing speed has been taken into account.

Turning to reading fluency of connected text, there is quite some research on the role of comprehension processes, and specifically vocabulary. Fuchs et al. (2001) argued that reading fluency of connected text is a good indicator of reading competence, because it involves all skills necessary for reading, including word recognition and comprehension skills (see also Samuels, 2006, 2007). Others have suggested that there may be a reciprocal relation between fluency and comprehension (e.g., Klauda and Guthrie, 2008; see also Jenkins et al., 2003; Lai et al., 2014), but further research is necessary to support this (Kuhn et al., 2010). Other studies on the role of text-level processes at the sentence level have mainly focused on reading comprehension as an outcome, instead of fluency (e.g., Foorman et al., 2015). There is also research on the influence of word-level reading fluency on sentence and text reading fluency (e.g., Schwanenflugel et al., 2004; Miller and Schwanenflugel, 2008; Benjamin and Schwanenflugel, 2010) as well as on reading comprehension (e.g., van Viersen et al., 2018). Yet, it is still unclear how individual differences in word-level reading processes (specifically word recognition speed and sequential processing efficiency) contribute to reading fluency of connected text, and in particular to sentence reading fluency. Moreover, information about the combined contributions of basic word- and text-level processes is also lacking. Sentence reading fluency is an interesting starting point in this respect, because it lies at the intersection between word-list reading fluency and (oral) text reading fluency. It could be considered as the fluency measure where word- and text-level processes first meet and is therefore included as one of the relevant reading fluency outcomes in this study.

The findings of one particular study on text reading fluency are also relevant for our understanding of sentence reading fluency: Altani et al. (2020) investigated word-level reading processes in text reading fluency and compared their results to those on serial word reading rate. Their brief texts were syntactically very simple and consisted of familiar (short and high frequency) words matched to those in the serial word-reading task. Their findings indicated some differences in terms of the contributions of the separate word-level processes. In English, sequential processing efficiency was the larger contributor to text reading fluency. In Greek, however, individual word recognition and sequential processing were equally strong predictors and explained equal amounts of total variance (see Zoccolotti et al., 2014, for similar findings using slightly different tasks in Italian Grade 6 children).

In addition, there are a few studies that focused on the combination of word- and text-level processes in text reading fluency. The results of Kim (2015) suggested that both vocabulary and syntactic skills were independent predictors of text reading fluency after controlling for sequential processing speed. However, this study was conducted in Korean kindergartners, hence very much beginning readers. Moreover, the findings were inconsistent over time (i.e., Kindergarten year 1 and Kindergarten year 2). Kim et al. (2011) found that word-list reading fluency of increasingly difficult words and listening comprehension (i.e., oral comprehension) together explain about 94% of the variance in text reading fluency of United States first graders (again, beginning readers). This is likely to be much less though when word-level processes underlying word-list reading fluency and text-level processes underlying listening comprehension are taken into account independently.

In this study, we aim to extend research into the mechanisms underlying reading fluency. To this end, we investigated the combined contribution of basic word- and text-level processes to a range of reading fluency measures that are assumed to differ in their underlying skill demands. The study is conducted with Dutch third grade children. Dutch is a semi-transparent language with a complex syllable structure (Seymour et al., 2003). Typical readers generally reach high accuracy levels by the end of second grade, after which fluency starts to increase rapidly (van Viersen et al., 2018). The third graders in our study can, on average, be considered intermediate-level readers. They have developed sufficient automaticity to show relevant variability in between-word processes and are able to free up enough cognitive resources to attend to comprehension aspects of reading. Hence our choice for this grade level given our range of fluency measures.

Several hypotheses can be formulated to gain more insight into the unique and shared contributions of individual word- and text-level processes to our set of reading fluency measures. First, we hypothesize that individual word recognition speed plays a crucial role in all fluency measures, but its individual contribution decreases with increasing complexity of the fluency measure. Second, we hypothesize that sequential processing speed will also be an independent predictor of all three fluency measures. However, if sequential processing speed represents both between- and within-word serial processes, its contribution could be larger to fluency measures in which words cannot be (fully) read by sight (i.e., word-list reading of increasingly difficult words). Third, we hypothesize that receptive vocabulary contributes to word-list reading fluency of increasingly difficult words as well as to sentence reading fluency. Syntactic skills are expected to only contribute to sentence reading fluency. A remaining question concerns the total amount of variance that can be explained by word- and text-level processes combined. Word-level processes are expected to take up the largest portion of variance in serial word reading rate, but it is not entirely clear whether they are similarly involved in word-list and sentence reading fluency. One possibility is that the additional involvement of text-level processes in these fluency measures accounts for additional variance. Alternatively, involvement of text-level processes could also result in a reduction of variance explained by word-level processes. Overall, these hypotheses are posited to reveal (a) the role of sequential processing speed across different reading fluency outcomes and (b) the point at which complexity and context become relevant for reading fluency to an extent that text-level factors come into play.

## Materials and Methods

### Participants

A total of 73 Dutch Grade 3 children (50.7% girls) participated in the study. Children came from four different schools in the middle and west of the Netherlands, recruited through school boards. Parents were informed about the school’s participation in the study and provided consent for their child to participate. Data were collected as part of a larger longitudinal study into orthographic learning (van Viersen et al., 2021) approved by the Ethics Committee of the University of Amsterdam (case no. 2017-CDE-8332). Children of all reading levels and language backgrounds participated in the study, but children with a dyslexia diagnosis or those who did not list Dutch as their preferred language were excluded. Background characteristics are provided in Table 1.

**TABLE 1:** Background characteristics.

Variable	*M*	*SD*	Min.	Max.
Age in months	106.22	4.81	97.00	116.00
Word reading fluency^a^	11.88	2.97	5.00	19.00
Receptive vocabulary^b^	105.51	11.04	74.00	128.00
Expressive grammar^a^	10.51	2.39	5.00	15.00

*^a^Standard score (M = 10, SD = 3).*

*^b^Standard score (M = 100, SD = 15).*

*See Methods for task descriptions.*

### Instruments

#### Individual Word Recognition

A discrete word-reading task was administered to measure individual word recognition speed. The task consisted of 36 high-frequency four-letter words previously used by van den Boer et al. (2016). Words were originally selected from the CELEX database (Baayen et al., 1993) and contained either vowel digraphs or consonant clusters (e.g., boer, vuur, stil, werk). The task was administered in DMDX (Forster and Forster, 2003) and was preceded by four practice items. Words were displayed one at a time in black 20-point Consolas on a white screen. Children had to read the word aloud when it appeared and their response was audio recorded. The experimenter controlled moving to the next item by pressing a key. Items were separated by an empty white screen. The raw score for discrete word reading was the mean reading time in seconds across correct items (including onset latency and articulation duration (see e.g., van den Boer and de Jong, 2015; Altani et al., 2018). Cronbach’s α was 0.96 on the current sample.

#### Sequential Processing

A serial digit-naming task was administered to measure sequential processing efficiency (Altani et al., 2018; Protopapas et al., 2018). A set of 36 digits, consisting of nine repetitions of four digits (i.e., 2, 3, 5, and 6), was displayed in four rows of nine items using DMDX. Children had to name the complete series of digits from the top left to bottom right as fast and accurately as possible. The task started with four practice items. The raw score for serial digit naming was the total naming time in seconds for the entire array, as is common for rapid automatized naming tasks (see also e.g., van den Boer and de Jong, 2015; Altani et al., 2018). Reliabilities of digit naming tasks lie between 0.79 and 0.87 in this age group (Evers et al., 2009–2012) and generally show high correlations with the same task in a somewhat different format (e.g., columns vs. rows; van den Bos et al., 2002).

#### Vocabulary

The *Peabody Picture Vocabulary Test NL* (PPVT-NL; Schlichting, 2005) was used to measure receptive vocabulary. Children had to choose the correct picture out of four alternatives to match a verbally presented target word. The test, consisting of 17 sets of 12 words, starts with the entry set that matches the child’s age. Correct answers are counted from the start set, which is the first set in which the child obtains at least four correct answers. The end set is the last set in which the child provides nine or more incorrect answers. The raw score is the number of correctly chosen pictures in the administered sets plus all non-administered items in preceding sets auto-scored as correct. Age-based standard scores are also available (*M* = 100, *SD* = 15). Reliability of the PPVT-NL has been evaluated as good (Egberink et al., 2017).

#### Syntactic Skills

The formulated sentences subtest of the *Clinical Evaluation of Language Fundamentals-4 NL* (CELF; Kort et al., 2010) was used to measure expressive grammar skills. Children had to make a sentence about a situation displayed in a picture using a verbally presented target word. For example, they had to use the word ‘*eindelijk*’ (finally) to formulate a grammatically correct sentence about a picture showing a boy handing in his homework (simpler item), or the words ‘*in plaats van*’ (instead) to describe a situation in which a boy chooses a book from a shelf (more difficult item). Quality of the formulated sentences was scored using the manual, which provided rules for the number of points awarded per sentence (ranging from 2 to 0). There were 20 items in total and testing was terminated after five consecutive sentences with zero points. Raw scores were used in the analyses. Age-based standard scores were also available. Internal consistency of the subtest is 0.78 (Evers et al., 2009–2012).

#### Serial Word Reading Rate

A serial word-reading task was administered to measure serial word reading rate (e.g., Protopapas et al., 2018). A set of 36 high-frequency four-letter words was displayed in four rows of nine words using DMDX. Words in this set were matched to those in the discrete word-reading task on onset phoneme, length, consonant-vowel structure, and frequency (van den Boer et al., 2016). Children had to read the words aloud from the top left to bottom right as fast and accurately as possible, starting with four practice items. The raw serial word-reading score for each child was the total reading time in seconds for the complete series of words (e.g., Altani et al., 2018).

#### Word-List Reading Fluency

The Dutch *Eén Minuut Test* (EMT; Brus and Voeten, 1999) was used to measure word-list reading fluency. Children had to read as many items as possible within 1 min. The test consisted of 116 items that increased in difficulty from one to four syllables. Raw score is the number of correctly read words within the time limit. Grade-level standard scores are available per semester (*M* = 10, *SD* = 3). Test–retest reliability is 0.90 (Evers et al., 2009–2012).

#### Sentence Reading Fluency

A measure of sentence reading fluency was obtained through a sentence-reading task in which children had to read aloud sentences displayed on a computer screen under eye tracking (see van Viersen et al., 2021). Eye movements were recorded in “remote” mode, without any form of head stabilization, allowing children to move freely within reasonable boundaries. The task contained 16 context-neutral sentences with similar structure (e.g., ‘*De groene rups at zijn buikje vol met blaadjes*’ [The green caterpillar filled his belly with leafs], ‘*Het gevlekte kalf sprong vrolijk door de wei*’ [The spotted calf jumped happily through the meadow]). The sentences were followed by a yes/no comprehension question (e.g., *Was het kalf buiten?* [Was the calf outside?]) to ensure that children were reading and not scanning. The sentences were part of a larger experiment containing an additional 64 sentences in which a target word was experimentally manipulated. Those sentences are not taken into account in the current study to avoid confounding effects of the manipulations. Children’s responses were recorded to determine reading times in seconds and number of errors per sentence (see below). This information was used to compute the mean number of correctly read words per second across all sentences.

### Procedure

Children were tested at school during two individual sessions in February and March 2019. The first session took about 20 min and contained the sentence-reading task. The second session contained the reading and related tasks used for the word- and text-level factors. This session was scheduled several days after the first session and lasted about 40 min. Testing was conducted by trained and supervised research assistants.

### Data Preparation

The voice recordings of the sentence-reading task were processed using CheckFiles 2.3.1 (distributed with CheckVocal; Protopapas, 2007). Vocal responses were displayed audiovisually (waveform and spectrogram) to allow marking offsets to determine the total reading time per sentence (including onset latency and articulation). Decoding errors were manually marked in a separate Excel file. Sentences with incomplete or missing vocal responses were discarded. The recorded responses from the discrete and serial reading and serial naming tasks were processed using CheckVocal 2.3.1 and 3.0a (Protopapas, 2007). Response times (RTs) were determined following the same procedure as described above. Errors were marked using the same software. RTs were converted to reading rates (i.e., number of items per second; see also Altani et al., 2017). For the discrete word-reading task, reading rates were averaged for each participant across correctly read words. For the serial naming and serial word-reading tasks, two different scores were calculated: *Rate* scores were computed through including correct *and* incorrect responses (i.e., 36 divided by the total RT), to match common practice in scoring serial naming tasks. In addition, *fluency* scores were computed through only including correct responses (i.e., number of correct items divided by total RT), thus penalizing decoding errors to match common practice in oral reading fluency measures (e.g., Altani et al., 2018; Protopapas et al., 2018).

### Analyses

The extent to which word- and text-level predictors explain variance in serial word reading rate, word-list reading fluency, and sentence reading fluency was assessed with a path model using *lavaan* (Rosseel, 2012) in R version 4.1.1 (R Core Team, 2021). This path model contains only observed variables and combines three multiple regressions that would otherwise have been conducted separately. Doing so allows us to take correlations between predictors as well as among outcomes into account. It also allow us to test for equality of regression coefficients among fluency outcomes. Rate variables (i.e., number of items per second) were preferred over fluency variables (i.e., number of correct items per second) for serial digit-naming and serial word-reading tasks. This approach matches previous studies using serial naming measures (see also Altani et al., 2020). Moreover, a direct comparison between models with reading/naming rate vs. reading/naming fluency scores for serial naming and word reading showed that they produced the same results. For word-list and sentence reading fluency, the number of correctly read words *per second* was used to match the scale of the serial and discrete tasks. The initial path model contained all three reading outcomes and their correlations, all four predictors and their correlations, and the intercepts of all predictors and outcomes. This comes down to a just-identified model (i.e., a model with zero degrees of freedom and perfect fit). Non-significant paths can be trimmed step-by-step to arrive at a more parsimonious solution and to allow for the evaluation of model fit. Exact model fit is evaluated using the χ^2^-value with associated *p*-value (non-significant *p*-value indicates good fit). Approximate model fit is evaluated using the root mean square error of approximation (RMSEA; good ≤ 0.05, acceptable ≤ 0.08) including 90% confidence interval (CI; not exceeding 0.10) and *p*_close_ (>0.05), comparative fit index (CFI; good ≥ 0.95, acceptable ≥ 0.90), and standardized root mean square residual (SRMR; good ≤ 0.05, acceptable ≤ 0.08; Kline, 2011; Little, 2013).

In addition, we performed a fixed-order regression analysis within SEM to examine the *unique* contributions of the word- and text-level predictors to the individual reading outcomes (see van den Boer et al., 2014; de Jong and van den Boer, 2021, for examples). In this analysis, the predictors were entered in the regression model in a pre-specified order, first word-level and then text-level predictors. Fixed-order regression with a SEM model requires the specification of so called phantom factors (de Jong, 1999; Macho and Ledermann, 2011). The phantom factors are uncorrelated latent variables with their variances fixed to one. The first phantom factor is identical to the predictor entered first in the regression model. The loading of the first predictor is set to one and its residual variance to zero. The second phantom factor captures the variance of the second predictor after the variance that this predictor has in common with the first has been removed. To this end, the loading of the second predictor on the first factor is allowed to vary freely, but its loading on the second phantom factor is fixed to one. As with the first predictor, the residual variance of the second predictor is specified to be zero. The same logic applies to the subsequent predictors that are included in the model. Thus, fixed-order regression in SEM requires an alternative specification of the relations among the predictors (see Figure 2). Subsequently, the proportion of variance explained by the first predictor in the first outcome variable is computed by squaring the correlation between the first phantom factor and this outcome variable. Squaring the, now partial, correlation between the second phantom factor and this outcome gives the additional variance explained by the second predictor controlling for the first predictor. The same logic applies to the third predictor. The square of the partial correlation between the fourth (and last) phantom factor and the outcome indicates the unique variance explained by the predictor entered in the fourth step while controlling for all other predictors in the model. This alternative model specification using phantom factors does not affect model fit (de Jong and van den Boer, 2021).

## Results

All variables were approximately normally distributed, based on examination of quantile-quantile plots and skewness and kurtosis indices. Three univariate outliers (i.e., based on *z*-score <−3.30 or >3.30 and scatterplots; one on word-list reading fluency, one on serial digit naming, and one on vocabulary) were winsorized (i.e., replaced with percentile-adjusted values) to decrease their influence. The proportion of missing data points across variables ranged from zero to 2.7%. Missing data were neither imputed nor excluded, as models were fit using full-information maximum likelihood estimation. This approach permits the inclusion of cases with missing data. The scores for vocabulary and syntactic skills were rescaled to ease the estimation procedure. Descriptive statistics of the variables used in the analyses are reported in Table 2.

**TABLE 2:** Descriptives for predictors and outcomes.

Variables	*N*	*M*	*SD*	Min.	Max.	Skew.	Kurt.
1. Discrete word reading^a^	72	0.96	0.13	0.70	1.27	0.36	−0.27
2. Serial digit naming^b^	73	1.65	0.31	1.08	2.35	0.11	−0.58
3. Vocabulary^c^	72	1.13	0.09	0.92	1.29	–0.29	−0.45
4. Syntactic skills^d^	72	2.57	0.44	1.50	3.40	–0.26	−0.65
5. Serial word reading rate^b^	71	1.74	0.35	0.81	2.51	–0.03	−0.06
6. Word-list reading fluency^e^	72	1.02	0.22	0.57	1.52	–0.00	−0.28
7. Sentence reading fluency^e^	71	1.98	0.40	1.00	2.86	–0.27	−0.30

*^a^Mean reading time in seconds across correctly named items.*

*^b^Rate (items per second).*

*^c^Raw score rescaled by dividing by 100.*

*^d^Raw score rescaled by dividing by 10.*

*^e^Fluency (correct items per second).*

Table 3 shows the correlations between all variables. Both discrete word reading and serial digit naming correlate moderately with all reading outcomes. Correlations between the text-level predictors and reading outcomes are not significant, except for the relation between vocabulary and sentence reading fluency and between syntactic skills and word-list reading fluency. In addition, correlations between serial word reading rate, word-list reading fluency, and sentence reading fluency are strong.

**TABLE 3:** Pearson’s correlations between predictors and outcomes.

Variables	1.	2.	3.	4.	5.	6.	7.
1. Discrete word reading	–						
2. Serial digit naming	0.25*	–					
3. Vocabulary raw score	–0.14	–0.22	–				
4. Syntactic skills raw score	0.05	–0.15	0.45***	–			
5. Serial word reading rate	0.41***	0.44***	0.02	0.00	–		
6. Word-list reading fluency	0.42***	0.32**	0.16	0.24*	0.70***	–	
7. Sentence reading fluency	0.33**	0.33**	0.25*	0.17	0.62***	0.75***	–

See Table 2 for variable units. *p < 0.05, **p < 0.01, ***p < 0.001.

**FIGURE 1:**
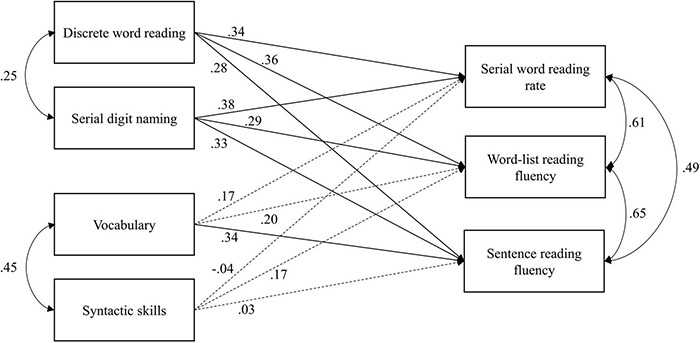
Path model of word- and text-level processes predicting reading fluency outcomes. Values on single-headed arrows are standardized regression coefficients. Double-headed arrows indicate correlations (standardized covariances). Solid lines indicate significant effects (*p* < 0.05) and dashed lines indicate non-significant effects. Error terms and correlations between predictors that are fixed at zero are not displayed to aid visibility.

The initial just-identified path model was trimmed by fixing non-significant correlations between word-level and text-level predictors at zero (four correlations in total). Correlations were fixed one at a time and the changes did not result in a significant deterioration in model fit. The final model, including the standardized path weights (i.e., β), is displayed in Figure 1. The fit of the final model was acceptable, χ^2^(4, *N* = 73) = 6.25, *p* = 0.18, RMSEA = 0.09, 90% CI = [0.00–0.21], *p*_close_ = 0.26, CFI = 0.99, SRMR = 0.07. Discrete word reading and serial digit naming both moderately predict all three reading outcomes and are weakly correlated with each other. Vocabulary only moderately predicts sentence reading fluency. Syntactic skills are not a significant predictor of any outcome, but do contribute to sentence reading fluency through their moderate correlation with vocabulary (specific indirect effect: β = 0.16, *p* = 0.02). Parameter estimates are listed in Supplementary Table 1. Combined, the word- and text-level processes account for 34.8% of the variance in serial word reading rate, 36.2% of the variance in word-list reading fluency, and 35.9% of the variance in sentence reading fluency.

Testing for possible differences between regression coefficients (as formulated in the hypotheses about the word-level processes) was done by constraining these coefficients to be equal across fluency outcomes for each individual word-level predictor. For the effect of discrete word reading, regression coefficients were found to be approximately equal across fluency measures. There was no significant deterioration in fit after posing equality constraints [i.e., Δχ^2^(2) = 2.87, *p* = 0.24]. For the effect of serial digit naming, regression coefficients were not found to be approximately equal, as constraining them resulted in a significant deterioration in model fit [i.e., Δχ^2^(2) = 8.97, *p* = 0.01]. Further examination revealed that the regression coefficient of serial digit naming on word-list reading fluency had to be estimated freely because it is significantly lower than the regression coefficients for serial word reading rate and sentence reading fluency. Further testing of differences between regression coefficients revealed that discrete word reading is a stronger predictor of word-list reading fluency than serial digit naming (*p* = 0.04).

**FIGURE 2:**
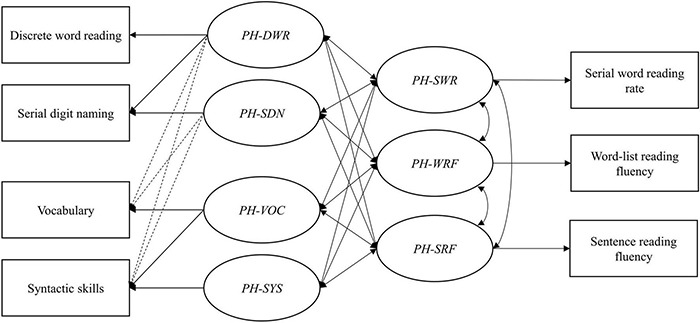
Fixed-order regression model predicting reading fluency outcomes from word- and text-level processes. PH, phantom factor; DWR, discrete word reading; SDN, serial digit naming; VOC, vocabulary; SYS, syntactic skills; SWR, serial word reading rate; WRF, word-list reading fluency; SFR, sentence reading fluency. Solid lines indicate freely estimated paths and dotted lines indicate paths that were fixed at zero. Error terms of observed variables and correlations between phantom factors that are fixed at zero are not displayed to aid visibility.

The variance contributed by each word- or text-level predictor separately was assessed through fixed-order regression in SEM using phantom factors for both the predictors and outcomes (de Jong, 1999; see Analyses for more details). The model with the phantom factors is presented in Figure 2. Factor loadings between predictors and predictor phantom factors were structured to correspond to sequential steps taken in a traditional hierarchical regression to determine the additional variance explained by each predictor, beyond variance accounted for by “previous” predictors. The order of the steps was determined by ranking the processes underlying reading fluency from most basic (i.e., individual word recognition speed) to more advanced (i.e., syntactic skills) based on theory (see also our hypotheses). Accordingly, discrete word reading was evaluated first. As can be deduced from Figure 2, PH-DWR contains all variance explained by discrete word reading. Serial digit naming was evaluated second. PH-SDN is the factor that remains after the variance that serial digit naming has in common with discrete word reading is accounted for. The same logic applies to vocabulary, which was evaluated third. Syntactic skills were evaluated last. As such, PH-SYS is the factor that remains after the all the other predictors have been controlled, that is, the unique variance explained by syntactic skills. The unique variance of the other predictors was determined by changing the order of the predictors through adaptation of the factor loadings to the phantom factors. Several factor loadings between observed predictors and predictor phantom factors were fixed at zero to mirror the correlations between the predictors in the initial path model (e.g., as vocabulary and discrete word reading did not correlate in the initial path model, the factor loading from vocabulary on the discrete word reading phantom factor is fixed at zero; see the dotted lines on the left in Figure 2).

The results of the fixed-order regression analyses are reported in Table 4. Discrete word reading accounts for a similar amount of (shared and unique) variance in all three fluency outcomes. The somewhat lower amount for sentence reading fluency is not likely to be significantly different based on the comparisons of regression coefficients in the original path model. Serial digit naming explains additional variance in all reading outcomes after discrete word reading is controlled and also contributes uniquely to all reading outcomes after controlling for all other predictors in the model. In line with the original path model, its contribution to word-list reading fluency seems lower than for the other fluency measures. Vocabulary explains additional variance in both word-list and sentence reading fluency, but only contributes uniquely to the latter. Syntactic skills do not explain any additional variance while controlling for the other predictors and do not contribute uniquely to any of the reading outcomes.

**TABLE 4:** Additional and unique variance (percent) per predictor for all reading outcomes.

Predictor	Serial word	Word-list	Sentence
	reading rate	reading fluency	reading fluency
**Additional variance**			
Discrete word reading	18.9***	18.4***	13.2***
Serial digit naming	13.6***	7.9**	10.1***
Vocabulary	2.2	7.5**	12.5***
Syntactic skills	0.1	2.4	0.1
*R* ^2^	34.8	36.5	35.9
**Unique variance**			
Discrete word reading	11.0***	12.0***	7.5**
Serial digit naming	13.6***	7.9**	10.1***
Vocabulary	2.2	3.0	9.2**
Syntactic skills	0.1	2.4	0.1

*p < 0.05, **p < 0.01, ***p < 0.001.

## Discussion

In this study, we investigated to what extent basic word- and text-level processes contribute to different measures of reading fluency to better understand the mechanisms underlying fluent reading. Individual word recognition speed and sequential processing efficiency were included as word-level processes, and vocabulary and syntactic skills were included as text-level processes. Reading rate of a list of unrelated familiar words (i.e., serial word reading rate), reading fluency of a list of increasingly difficult words (i.e., word-list reading fluency), and reading fluency of sentences were assessed as reading fluency measures. These measures represent a relevant variation in task complexity and availability of context. The main findings indicate that sequential processing efficiency plays an essential role in reading fluency across reading fluency measures besides individual word recognition speed. In addition, text-level processes come into play when complexity of fluency measures increases and context becomes available. However, the exact timing of these effects requires further investigation. The total variance that the word- and text-level factors accounted for did not increase appreciably with increasing complexity of fluency tasks or availability of context (all *R*^2^ within 0.015 of one another).

### Word-Level Processes

The first hypothesis regarded the crucial role of individual word recognition speed as a basic word-level process underlying reading fluency across measures varying in complexity and availability of context. This was largely confirmed, as individual word recognition speed indeed contributed uniquely to every fluency measure. This finding confirms previous research on the role of individual word recognition in serial word reading rate (Protopapas et al., 2013; Altani et al., 2017) and word-list reading fluency (de Jong, 2011). However, its individual contribution did not decrease with increasing complexity of the fluency measures. Additionally, individual word recognition speed was found to account for a similar amount of variance in serial word reading rate and sentence reading fluency as sequential processing efficiency, which was hypothesized as another essential word-level process. This latter finding provides further evidence that individual word recognition speed is not the sole factor necessary and sufficient for fluent reading of unrelated words in lists. This can now be extended to words in connected text as well (see also Altani et al., 2020).

The second hypothesis positing sequential processing efficiency as an additional independent word-level predictor of reading fluency was confirmed. Sequential processing efficiency was indeed found to be a unique predictor of all three fluency measures. However, the size of its individual contribution differed across fluency measures. The amount of variance sequential processing efficiency explained in word-list reading of familiar words was similar to findings of previous studies covering serial word reading rate (e.g., van den Boer et al., 2016; Altani et al., 2017, 2018; see also de Jong, 2011; van den Boer and de Jong, 2015; Altani et al., 2020). However, in contrast to our hypothesis, its predictive value was found to decrease when moving from serial word reading to word-list reading fluency. This was also reflected in a lower amount of unique and shared variance in word-list reading fluency accounted for by sequential processing efficiency. In other words, sequential processing efficiency seems to become less important when words are less likely to be read by sight. This does not align with the suggestion that sequential processing efficiency might account for both between- and within-word serial processes, and would thus contribute more variance when words cannot be (fully) read by sight in more complex fluency measures (de Jong, 2011; van den Boer et al., 2016). The correlations reported by de Jong (2011) suggest that sequential processing efficiency should at least explain comparable amounts of unique variance in both word-list reading measures. As our study is the first to compare the individual contributions of word-level processes to reading fluency of word lists of familiar and increasingly difficult words, replication is warranted. Yet, our findings add to the ongoing debate about the role of sequential processing efficiency in reading fluency. They also highlight the need to determine whether within- and between-word serial processes are partly overlapping constructs or completely separate skills (see also de Jong and van den Boer, 2021).

Regarding sentence reading fluency, the role of sequential processing efficiency seems to increase again (to a level comparable with that in serial word reading rate) when words are no longer unrelated but interconnected through supra-lexical elements. The contribution of sequential processing efficiency is similar to that of individual word recognition speed. This aligns with the findings of Altani et al. (2020) on Greek third graders. The specific amount of variance explained by sequential processing efficiency in that study was comparable with our finding and about equal to the amount explained by individual word recognition speed. In contrast, sequential processing efficiency was the dominant word-level process predicting reading fluency of texts containing simple short words in English-speaking third graders. As stated before, Dutch is a semi-transparent language that is somewhere in between the more transparent Greek and more opaque English. These findings are consistent with the idea that languages differ in the relative importance of word-level processes for reading fluency (Altani et al., 2017, 2020). Overall, our findings for sequential processing efficiency support the suggestion that reading fluency is further developed by the coordination of processing across multiple items in a sequence once sight word reading has been established (Protopapas et al., 2013, 2018).

### Text-Level Processes

The third hypothesis, regarding the contribution of vocabulary to both word-list reading fluency and sentence reading fluency, and the contribution of syntactic skills solely to sentence reading fluency, was partly confirmed. As expected, vocabulary accounted for unique variance in sentence reading fluency. In contrast, syntactic skills did not contribute to sentence reading fluency, despite a small indirect effect through vocabulary. This is the first study assessing this specific set of word- and text-level factors in reading fluency of sentences. Therefore, it is reassuring that the findings align with previous studies on the interaction between word reading and semantics in the presence of context (e.g., Nation and Snowling, 1998; Jenkins et al., 2003; Kim et al., 2014). However, the absence of an influence of syntactic skills stands in contrast to studies illustrating the essential role of syntax as a supra-lexical influence on reading fluency of connected text (e.g., Mokhtari and Thompson, 2006; van den Bosch et al., 2018).

An explanation might be that the sentences used in the sentence-reading task of this study were relatively simple. All sentences followed a similar structure and were of limited length (*M* = 8.4 words, range 7–14). As a comparison: the study by van den Bosch et al. (2018) included sentences with strong manipulations in syntactic complexity through presence and absence of connectives (i.e., ‘because’) as well as linear order of clauses (cause-effect vs. effect-cause). Their study also focused on second language learners with limited syntactic skills. Our measure of expressive grammar specifically targeted aspects deemed relevant for sentence reading fluency, challenging children to formulate sentences using increasingly complex connectives in specific contexts. An expressive measure was preferred over a receptive measure, such as sentence repetition from the CELF test battery (Kort et al., 2010), to ensure sufficient variability in scores across our sample of typically developing readers. However, the syntactic complexity of the sentences in our experiment may not have been high enough to require such substantial syntactic processing. Accordingly, sentence complexity did not reach the basic threshold to elicit effects of syntax on sentence reading fluency.

In that respect, the findings for the role of vocabulary in word-list reading fluency of increasingly difficult words might be explained through a similar argument. In this fluency measure, vocabulary accounted for additional variance after controlling for word-level processes, but did not account for any unique variance. The nature of the items in this task is quite difficult to capture and not as straightforward as in the word-list reading measure with short and familiar words. Words in the word-list reading fluency task increase in length (number of letters and syllables) and difficulty (complexity of syllables). At the same time, they also decrease in frequency. Although the least familiar, most complex items may not have been read by sight, children may not have reached this point during the time limit of the task. Consequently, they may not have needed their vocabulary knowledge yet to support their word identification skills.

Taken together, these findings imply that systematic manipulation of complexity, in terms of demands on both word-level processes and text-level processes, is necessary to acquire more knowledge about interactions between these processes during fluent reading. It then becomes important to determine *when* text-level processes come into play once meaning plays a role or words are connected through supra-lexical elements. Likewise, the type of text-level factors might also be important. For example, morphological skills may influence reading fluency more at the within-sentence level or when increasing difficulty of words also reflects higher morphological complexity (see e.g., Berninger et al., 2001). Syntactic skills may account for more variance at the between-sentence level. Further systematic manipulation and comparison of reading fluency measures and relevant factors is thus warranted.

### Reading Fluency Measures

The remaining question regards the total amount of explained variance and the relative importance of each word- and text-level process in the individual fluency measures. This question was partly answered: Individual word recognition speed and sequential processing efficiency both have an important role in all three reading fluency measures. But their combined impact slightly decreases with increasing complexity of fluency measures. Yet, the additional influence of text-level processes in more complex reading fluency measures does not lead to a higher total amount of explained variance. Our specific set of word- and text-level processes accounts for about the same amount of variance in all reading fluency measures included in the study. It seems that the addition of text-level processes, that is, mainly vocabulary, leads to a redistribution of variance across the different predictors. This then results in a reduction of variance accounted for by word-level processes. This might also partly explain why sequential processing efficiency seems to play a smaller role in word-list and sentence reading fluency than in serial word reading, to which vocabulary did not contribute in any way. Still, the underlying mechanisms might be different for word-list and sentence reading fluency (see above). Although text-level processes may be expected to account for additional variance on top of basic word-level processes, they only appear to fill the resulting gap when word-level processes become somewhat less important. This leads to similar amounts of total explained variance across fluency measures of varying complexity.

Turning to each separate reading fluency measure, our findings for serial word reading rate in Dutch children are again somewhere in between those for Greek and English children of the same age (third graders) reported by Altani et al. (2020). Individual word recognition speed and sequential processing efficiency seem to be the two main reading processes underlying word-list reading of familiar words. Sequential processing efficiency accounts for a similar amount of unique variance as individual word recognition, in alignment with the pattern for Greek children. It is surprising that the dominance of sequential processing efficiency as a word-level process is not yet more visible in the Greek children in the Altani et al. (2020) study and the Dutch children in our study. Fluency-related processes generally develop earlier in more transparent languages. The amounts of unique variance accounted for by the individual word-level factors in our study are also more in line with the findings in Greek children. What further stands out is that the total amount of variance that is explained by the word-level processes is considerably lower in our study (32.5%) than the 49–50% in both Greek and English children reported by Altani et al. (2020). This is unexpected because the lower correlation between word-level predictors in Dutch compared to Greek and English (i.e., 0.25 vs. 0.44 and 0.36) suggest lower shared variance between the two word-level predictors in Dutch. This would normally result in higher total amounts of explained variance, as the two processes account for different sources of variance. The amounts of explained variance may be higher for both other languages due to the use of bisyllabic words in Greek (which may be processed in larger chunks instead of letter-by-letter) and whole-word reading in English. As Dutch is more transparent than English, but used shorter words than Greek (i.e., monosyllabic words), this results in lower overall shared variance. It is not clear why both processes are then not better able to predict different components of variance in reading fluency. More cross-language comparisons, such as done by van den Boer et al. (2016) and Altani et al. (2017, 2020), are needed to gain more insight into how orthography influences the role of word-level processes. Such studies can also shed more light on the weight of word-level processes across different types of fluency measures, as well as the timing of developmental shifts in dominance of specific processes.

Word-level processes (especially sequential processing efficiency) seem to contribute less to word-list reading fluency than to the simpler measure of serial word reading. The influence of sequential processing efficiency would be expected to increase as fluency tasks become more complex and entail both between- and within-word serial processes (de Jong, 2011; van den Boer et al., 2016); this pattern is not borne out in our data. Instead, it seems that the role of retrieval processes (as captured by the discrete word-reading task aiming to measure sight word reading) is more or less similar across all three fluency measures. Consequently, sequential processing efficiency may only account for the between-word processing and not for within-word processes. Additional factors related to task difficulty may be relevant here: The mean number of words processed per second is much higher for serial word reading (1.74) than for word-list reading fluency (1.02). This makes between-word serial processing much more prominent in the former task.

No previous studies have examined the prediction of sentence reading fluency by word- and text-level processes. There is thus no baseline against which we can compare the proportion of variance in sentence reading fluency that was accounted for in our study. Studies including listening comprehension or word-list reading fluency as predictors of text reading fluency (e.g., Kim et al., 2011) are not directly relevant for this comparison. These more encompassing tasks do not provide the level of detail in terms of underlying mechanisms associated with fluent reading that we aimed to identify in our study. As such, including a wider but also more controlled range of fluency measures is essential for unraveling the underlying processes that are involved in reading fluency development and that may be deficient in struggling readers. Systematic manipulations should entail both word- and text-level demands.

### Implications

Our findings confirm that reading fluency is more than just fast recognition of individual words. Reading fluency of word lists, as so often used in clinical and educational contexts, also requires the coordination of processes across consecutive words. Moreover, reading fluency of connected text, which would be a more ecologically valid measure of reading fluency, also requires the integration of text-level comprehension processes. Even though we are only beginning to understand how these individual processes interact and contribute to reading fluency, this study provides important clues for future research. We were able to illustrate that there are meaningful differences between fluency measures concerning the underlying processes that play a role in them. If we want to understand where these differences come from and how they can be explained (and perhaps eventually supported), we need to start mapping underlying processes more systematically to identify potential bottlenecks during development. Aspects to take into account pertain to (1) creating relevant and systematic variation in demands on word- and text-level processes within fluency measures, (2) ascertain variation in complexity and length of materials that match reading fluency across development (e.g., adding pseudoword-list reading, syntactically complex sentences, and short and longer texts), (3) inclusion of a broader range of word- and text-level processes (e.g., adding decoding and morphological skills), (4) more detailed tracking of the timing of developmental shifts in underlying processes, and (5) possible effects of orthography (e.g., van den Boer et al., 2016; Altani et al., 2017, 2020).

Regarding the relevance for diagnostic practice, our findings confirm that typical word reading efficiency tests (such as TOWRE; Torgesen et al., 2012) are very suitable as a screening and diagnostic tool for word-level reading difficulties: they seem to capture the main mechanisms underlying word reading fluency (Protopapas et al., 2018). Our study further shows that this measure captures both individual word recognition speed and sequential processing efficiency, as well as some overall word knowledge. These tests provide a good indication of a child’s word-level reading fluency skills when combined with a pseudoword-list reading fluency test, filtering out the role of vocabulary. However, we contend that it is important to increase both the breadth and depth of reading fluency assessment, especially in developing readers, if we want to understand what part of reading fluency may be particularly weak or underdeveloped. By depth we mean specific assessment of underlying word- and/or text-level processes (e.g., individual word recognition and sequential processing; see e.g., Nomvete and Easterbrooks, 2020, for text-level suggestions) with the aim to identify specific deficiencies. By increasing breadth we mean mapping reading fluency through measures that have educational and societal relevance (i.e., covering connected text) and fit literacy progress as reading becomes more advanced, with the aim to make instructional decisions (see Washburn, 2022, for an overview). Combined, the resulting information can provide input for further improvement of literacy instruction throughout education and foster tailored assessment and interventions for struggling readers.

## Data Availability Statement

The original contributions presented in the study are publicly available. These data can be found here: https://osf.io/mkurw/?view_only=51d0227d5df74e229f1e0c14b8cb3047.

## Ethics Statement

The studies involving human participants were reviewed and approved by The ethics committee of the University of Amsterdam (case number 2017-CDE-8332). Written informed consent to participate in this study was provided by the participants’ legal guardian/next of kin.

## Author Contributions

SV acquired funding, conceptualized and managed the study, developed the measures, collected the data, conducted the analyses, interpreted the findings, and wrote and revised the manuscript as lead author. AP contributed to the conception and design of the study, the data processing, analyses and interpretation of the findings, and reviewed and edited the manuscript. PJ contributed to acquiring funding and conceptualizing the study, analyses and interpretation of findings, and reviewed and edited the manuscript. All authors contributed to the article and approved the submitted version.

## Conflict of Interest

The authors declare that the research was conducted in the absence of any commercial or financial relationships that could be construed as a potential conflict of interest.

## Publisher’s Note

All claims expressed in this article are solely those of the authors and do not necessarily represent those of their affiliated organizations, or those of the publisher, the editors and the reviewers. Any product that may be evaluated in this article, or claim that may be made by its manufacturer, is not guaranteed or endorsed by the publisher.
