# Supplementary material for: Word- and Text-Level Processes Contributing to Fluent Reading of Word Lists and Sentences
Source: Front Psychol. 2022 Jan 10;12:789313. doi: 10.3389/fpsyg.2021.789313 (PMC8784374; doi:10.3389/fpsyg.2021.789313)
Supplement: Supplementary file 1 [file Table_1.pdf]

Word- and Text-level Processes Contributing to Fluent Reading of Word Lists and Sentences

Sietske van Viersen, Athanassios Protopapas, & Peter F. de Jong

Supplementary Table 1 *Parameter Estimates of the Path Model Reported in Figure 1*

Parameter	Unstandardized	*SE*	*p*	Standardized
Regressions				
Serial word reading rate				
Discrete word reading	0.94	0.27	.001	.34
Serial digit naming	0.43	0.12	< .001	.38
Vocabulary	0.64	0.42	.134	.17
Syntactic skills	−0.03	0.09	.715	−.04
Word-list reading fluency				
Discrete word reading	0.63	0.17	< .001	.36
Serial digit naming	0.21	0.07	.004	.29
Vocabulary	0.48	0.27	.071	.20
Syntactic skills	0.09	0.05	.104	.17
Sentence reading fluency				
Discrete word reading	0.89	0.31	.004	.28
Serial digit naming	0.43	0.13	.001	.33
Vocabulary	1.52	0.49	.002	.34
Syntactic skills	0.03	0.10	.792	.03
Covariances				
Discrete word reading				
Serial digit naming	0.01	0.01	.044	.25
Vocabulary	0.00			.00
Syntactic skills	0.00			.00
Serial digit naming				
Vocabulary	0.00			.00
Syntactic skills	0.00			.00
Vocabulary				
Syntactic skills	0.02	0.01	< .001	.45
Serial word reading rate				
Word-list reading fluency	0.03	0.01	< .001	.61
Sentence reading fluency	0.05	0.01	< .001	.49
Word-list reading fluency				
Sentence reading fluency	0.04	0.01	< .001	.65
Intercepts				
Discrete word reading	0.96	0.02	< .001	7.47
Serial digit naming	1.65	0.04	< .001	5.35
Vocabulary	1.13	0.01	< .001	12.36
Syntactic skills	2.57	0.05	< .001	5.84
Serial word reading rate	−0.51	0.56	.366	−1.45
Word-list reading fluency	−0.69	0.35	.052	−3.07
Sentence reading fluency	−1.37	0.65	.034	−3.37

Variances				
Discrete word reading	0.02	0.00	< .001	1.00
Serial digit naming	0.10	0.02	< .001	1.00
Vocabulary	0.01	0.00	< .001	1.00
Syntactic skills	0.19	0.03	< .001	1.00
Serial word reading rate	0.08	0.01	< .001	.652
Word-list reading fluency	0.03	0.01	< .001	.638
Sentence reading fluency	0.11	0.02	< .001	.641
R^2				
Serial word reading rate	0.348			
Word-list reading fluency	0.362			
Sentence reading fluency	0.359			
